# System for Patterning Polydopamine and VAPG Peptide on Polytetrafluoroethylene and Biodegradable Polyesters for Patterned Growth of Smooth Muscle Cells In Vitro

**DOI:** 10.1021/acsomega.3c02114

**Published:** 2023-06-05

**Authors:** Kamil Kopeć, Rafał Podgórski, Tomasz Ciach, Michał Wojasiński

**Affiliations:** † Warsaw University of Technology, Faculty of Chemical and Process Engineering, Department of Biotechnology and Bioprocess Engineering, Waryńskiego 1, 00-645 Warsaw, Poland; ‡ Warsaw University of Technology, CEZAMAT, Poleczki 19, 02-822 Warsaw, Poland

## Abstract

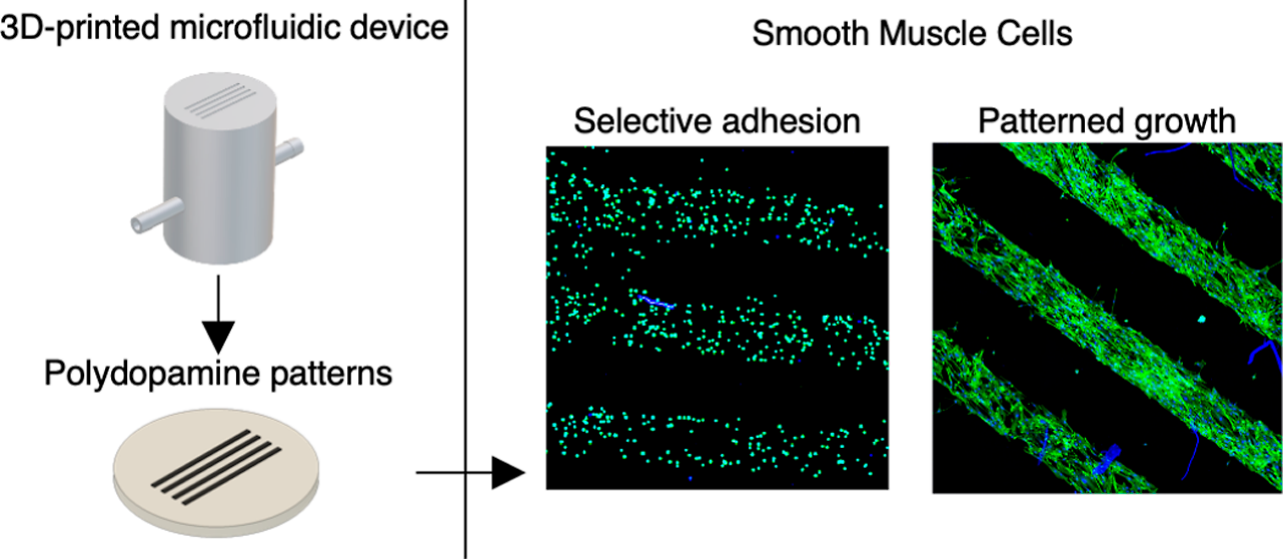

Biomaterial’s surface functionalization for selective adhesion and patterned cell growth remains essential in developing novel implantable medical devices for regenerative medicine applications. We built and applied a 3D-printed microfluidic device to fabricate polydopamine (PDA) patterns on the surface of polytetrafluoroethylene (PTFE), poly(l-lactic acid-*co*-D,l-lactic acid) (PLA), and poly(lactic acid-*co*-glycolic acid) (PLGA). Then, we covalently attached the Val-Ala-Pro-Gly (VAPG) peptide to the created PDA pattern to promote the adhesion of the smooth muscle cells (SMCs). We proved that the fabrication of PDA patterns allows for the selective adhesion of mouse fibroblast and human SMCs to PDA-patterned surfaces after only 30 min of in vitro cultivation. After 7 days of SMC culture, we observed the proliferation of cells only along the patterns on PTFE but over the entire surface of the PLA and PLGA, regardless of patterning. This means that the presented approach is beneficial for application to materials resistant to cell adhesion and proliferation. The additional attachment of the VAPG peptide to the PDA patterns did not bring measurable benefits due to the high increase in adhesion and patterned cell proliferation by PDA itself.

## 1 Introduction

Cell cultures are widely employed in biomedical research, especially in drug discovery and tissue engineering, as a simple model system for studying processes in human tissues. In traditional cultures, cells are propagated on flat surfaces of culture vessels in a spatially uncontrolled manner, which does not accurately reflect the behavior of cells in tissues. In natural biological systems, complex tissue microenvironment gives physical and topographical stimuli that influence many cell morphogenetic processes.^1−3^ Cells usually lose their phenotype in the simplified conventional culture system, where the surface of a plastic vessel serves as a cell substrate.^4^ Furthermore, the natural development of tissue is preceded by the controlled generation of organized cell patterns,^5^ which is impossible in standard cultures. One approach to overcome these limitations relies on physical or chemical modification of the surface of materials to stimulate cells to grow in a targeted manner according to the designed structure.

Micropatterning is a widely investigated method for the targeted growth of animal cells on the surface of polymeric materials. This technique allows for the cultivation of cells that are appropriately elongated and organized in aligned structures, which is a representation closer to the natural environment and morphology of certain tissues in situ than traditional culture methods on flat and extensive surfaces of standard culture vessels.^6^ Such organized culture can be achieved by seeding cells onto topographically patterned surfaces that physically direct cell growth, onto chemically patterned surfaces that alter cell adhesion in selected substrate regions, or by targeted delivery of cells to selected substrate regions.^7^ The most commonly used micropatterning techniques in tissue engineering are photolithography, electron beam lithography, soft lithography, microcontact printing, and microfluidic patterning.^7,8^ Depending on the scale, micropatterning can be used to study the impact of geometric constraints on a single cell, single populations generating cellular microsheets, or tissues containing micro-scale patterns of multiple cell populations.^9^ Patterned cell cultures can be used, for instance, to study surface cell adhesion^10^ and cell migration,^11^ or as model tissues to study the infectivity of pathogens^12^ and the effectiveness and safety of various drugs.^13−15^

Micropatterning is a technique instrumental in tissue engineering for regenerative medicine. An important goal in this area of science is to regulate the alignment of cells in tissues being regenerated in a controlled microenvironment.^16^ This should be achieved on the surface of biocompatible or biodegradable grafts, prostheses, or scaffolds used to repair or regenerate damaged tissues.^17^ The guided alignment of the cells is critical in cultures of muscle and nerve cells, for which biological functionality strongly depends on the proper organization in tissues, which is precisely regulated in organisms.^18−21^

Topographical and chemical micropatterning has been used for the targeted growth of muscle cells. Huang et al.^22^ used micropatterned polymer membranes of PDMS with parallel microgrooves to align murine C2C12 myoblasts growth. The same cell line was cultivated by Bettadapur et al.^23^ on micromolded gelatin hydrogels cross-linked with microbial transglutaminase, which allowed us to maintain aligned skeletal structures of myotubes in culture. Fujie et al.^24^ presented a different approach to topographic micropatterning for muscle cell culturing. They produced microfabricated poly(lactic acid-*co*-glycolic acid (PLGA) nanoribbon sheets composed of aligned PLGA nanoribbons connected and fixed by external strips of the same polymer. Such a structure facilitated the alignment of C2C12, which was directed by the aligned direction of nanoribbons. Ebrahimi et al.^25^ fabricated gelatin methacryloyl hydrogel fibers with engraved longitudinal microgrooves on the surface. They have shown that combining topographical cues from surface micropatterning and biochemical stimulation promoted the alignment of C2C12 myoblasts and augmented myotube formation during differentiation. Chemical micropatterning was used by Yu et al.^26^ by coating glass with a uniform layer of poly(ethylene glycol) (PEG) with a density gradient of azide groups. They covalently attached the Val-Ala-Pro-Gly (VAPG) peptide to azide groups which resulted in selective enhancement of the adhesion and mobility of human vascular smooth muscle cells (SMCs) on the VAPG density gradient and directional migration toward the higher peptide density. As one more example, Wu et al.^27^ introduced methoxy PEG chemical striped patterns with the density gradient in a parallel direction on the surface of the glass, which allowed us to direct the orientation and migration of human vascular SMCs.

Polydopamine (PDA) is a biomimetic polymer with the potential for use in chemical patterning to enhance adhesion and guide cell growth. PDA firmly adheres to almost all solid surfaces, regardless of the surface chemistry, creating a thin biocompatible and hydrophilic layer.^28^ PDA is known to promote the adhesion and growth of many mammalian cells, making it attractive for application in tissue engineering.^29^ Furthermore, the PDA layer can be post-functionalized by covalent attachment of molecules containing amine or thiol groups via Michael addition or Schiff base reaction. This allows the fabrication of functional layers with grafted biomolecules, e.g., proteins, peptides, and growth factors.^30^ In our previous work, we used PDA to coat a polytetrafluoroethylene (PTFE) vascular prosthesis and attach gelatin to its surface, which resulted in full endothelialization of this prosthesis in 3 days.^31^ Other authors have used PDA to attach extracellular matrix-derived cell adhesion peptides, such as RGD,^32^ REDV,^33^ and YIGSR.^34^ PDA-patterned substrates have already been studied for selective adhesion and targeted cell growth. Chien et al.^35^ used microcontact printing to fabricate PDA patterns on the surface of glass, silicon, gold, polystyrene, and PEG. They proved that L929 mouse fibroblasts selectively adhere to PDA patterns. Ku et al.^36^ patterned PDA on the PDMS surface by the microfluidic method and obtained selective adhesion and alignment in the direction of striped PDA patterns of different cells: fibrosarcoma HT1080, mouse preosteoblast MC3T3-E1, and mouse fibroblast NIH-3T3.^36^ However, these studies do not provide knowledge of applying PDA patterns to selective adhesion and patterned growth of muscle cells.

In this study, we designed and built a 3D-printed device for microfluidic patterning of a PDA for selective cell adhesion and patterned cell growth. We applied our device to fabricate PDA patterns on the surface of PTFE, which is a biocompatible, resistant to cell adhesion polymer clinically used as a soft tissue implant material,^37,38^ as well as on poly(l-lactic acid-*co*-D,l-lactic acid) (PLA) and PLGA, that are biodegradable synthetic materials widely studied for applications in regenerative medicine.^39^ We covalently attached the elastin-derived peptide VAPG to the PDA patterns. The VAPG peptide is a biospecific cell adhesion ligand for SMCs. It binds to these cells via the cell surface receptors.^26^ The attachment of this particular peptide to PDA is yet to be studied so far. Therefore, we decided to investigate the effect of PDA patterns with VAPG on the adhesion and proliferation of SMCs. We performed experiments to answer the thesis of whether PDA and VAPG can be used in systems that aim to create patterns on polymers that will support SMC adhesion and growth.

## 2 Experimental Section

### 2.1 Materials and Chemicals

PTFE fluoroplast-4 tape was purchased from HaloPolymer (Russia). Biomedical grade PLA (70:30) Resomer LR 706 S and biomedical grade PLGA (85:15) Resomer LG 855 S were purchased from Evonik Industries (Germany). Polypropylene (PP) foil was purchased from Tuplex (Poland). Dopamine hydrochloride (99%) was purchased from Alfa Aesar (Germany). Dichloromethane (99.5%) was purchased from Linegal Chemicals. Sodium periodate (≥99.8%), acetic acid (≥99%), sodium acetate (≥99%), sodium carbonate (≥99.5%), sodium bicarbonate (≥99%), bovine serum albumin (BSA), Triton X-100 (≥99%), and paraformaldehyde (PFA) (≥95%) were purchased from Sigma-Aldrich (Germany). Val-Ala-Pro-Gly (VAPG) peptide (>98%) was synthesized and delivered by Novazym (Poland). The murine fibroblast cell line (L929) was purchased from Sigma-Aldrich, and Human Coronary Artery SMCs and Smooth Muscle Cell Growth Medium 2 kit were purchased from PromoCell. Dulbecco’s Modified Eagle’s Medium (DMEM), Dulbecco’s phosphate-buffered saline (DPBS), fetal bovine serum (FBS), penicillin (10,000 U·mL^–1^), and streptomycin (10,000 μg·mL^–1^) solution, Trypsin/EDTA solution, CyQUANT XTT cell viability assay (XTT), Alexa Fluor 488 Phalloidin, and 4′,6-diamidino-2-phenylindole dihydrochloride (DAPI) were obtained from ThermoFisher Scientific. The water used in experiments was purified by a Milli-Q water system (Merck, Germany).

### 2.2 Device for PDA Patterning

The 3D model of the device for PDA pattering was designed in AutoCAD 2016 (Autodesk, USA). The model design was based on a cylinder shape with a diameter of 16 and 22.44 mm of height, inlet and outlet (2.5 mm) connector nozzles, and four parallel, 400 μm wide microchannels on the working surface—the patterned microfluidic mask. The housing base model for covering the device’s working surface was designed as a 36 mm in diameter and 10 mm in height cylinder, with a cylindrical cavity in the center of 16.2 mm in diameter and 5 mm in depth. 3D models were exported as STL files and imported into PreForm software (Formlabs, USA). The imported models were tilted at an angle of 60° to the build platform, and model supports were made by an “auto-generate all” tool. The thickness of each layer of the 3D models was set to 50 μm, and a Clear v4 resin (Formlabs, USA) was selected as the printing material. The prepared models were sent to the Form 3 3D printer (Formlabs, USA), where the 3D-printing process occurred. After 3D printing, the obtained device was rinsed for 10 min in isopropanol. The surfaces and channels of the device were blown through with compressed air, and finally, the cleaned and dried device was put into the Form Cure (Formlabs, USA) chamber, where it stayed for 30 min, cured with UV radiation, and heated up to 60 °C. The patterned microfluidic mask of the cured device was polished with sandpaper with 1200 gradation, washed with water, and dried with compressed air.

### 2.3 Substrate Preparation

PTFE foil was used without pretreatment. For PLA and PLGA foil preparation, 2.5 g of each polymer was dissolved in 22.5 mL of dichloromethane and mixed overnight with a magnetic stirrer. The prepared solutions were dispensed on a PP sheet with an Elcometer 3700 device set to 1 mm. The PP sheet was used to prevent curling up PLA or PLGA foils after drying. Poured materials were dried overnight at 50 °C, and dried foils were cut into discs with a diameter of 14 mm or stripes with a size of 5 × 100 mm.

### 2.4 PDA Patterns and PDA Patterns + VAPG Preparation

PDA patterns were produced using the device described above. Discs of tested materials with a diameter of 14 mm were placed on the patterned microfluidic mask, covered with the rubber base, and closed with the housing base. The housing base was pressed against the patterned microfluidic mask by a clamp. The PDA layer was formed on the polymer surface in the designed pattern by the oxidative polymerization of dopamine in a reaction for which the effective reagent concentration and the pH value were established in a previous study.^40^ Dopamine polymerization was started by mixing 4 mL of each of two solutions: (i) 4.0 mg·mL^–1^ (w/v) dopamine hydrochloride, and (ii) 2.8 mg·mL^–1^ (w/v) sodium periodate in a 10 mM acetate buffer at pH 5.5. Immediately after mixing, 5 mL of the polymerization solution was put in the syringe. The syringe was placed in the syringe pump, which dispensed the solution into the inlet channel of the device and further into the microchannels of the patterned mask, where the solution was in contact with the surface of the modified materials. The patterns production was carried out at a flow rate of 0.1 mL·h^–1^ for 40 min at room temperature. After this process, the PDA-patterned samples were removed from the device, rinsed intensively in water, and dried at 50 °C for 30 min.

The VAPG peptide was attached to PDA patterns by immersing the samples of PDA-patterned substrates in a 15 μM peptide solution in 0.1 M carbonate buffer at pH 9.5 for 24 h at room temperature in the dark. After the reaction, the PDA-patterned + VAPG samples were rinsed intensively in water and dried at room temperature in the dark.

### 2.5 Stereomicroscopy

The width of the PDA patterns, hereafter referred to as the patch size, was measured using the stereoscopic microscope Leica M205 C (Leica Microsystems GmbH, Germany). Analyzes were performed using Leica Application Suite software (Leica Microsystems GmbH, Germany). Three independent samples with four patterns for each sample were analyzed, and the path size was measured in three places for each pattern. The results are presented as mean ± SD (*n* = 36).

### 2.6 Attenuated Total Reflectance-Fourier Transform Infrared (ATR-FTIR) Spectroscopy

The qualitative efficacy of producing PDA patterns was analyzed by ATR-FTIR spectroscopy using a spectrometer Nicolet 6700 (Thermo Scientific, Germany). The spectra were detected in the attenuated total reflection (ATR) mode and analyzed with OMNIC 8.3 software (Thermo Scientific, Germany). One characteristic spectrum for each tested material was selected for presentation.

### 2.7 Water Contact Angle Measurement

The tested materials’ water contact angle (WCA) was measured by the sessile drop method using a goniometer DSA100 (Krüss GmbH, Germany). 1 μL of water droplets was dispensed on the tested materials, and the WCA values were measured using Advance 1.4.1.2 software (Krüss GmbH, Germany). For samples with PDA and PDA patterns + VAPG, a drop of water was placed directly on the pattern. The goniometer camera was positioned perpendicular to the length of the pattern (see Figure 2B). Four independent samples were tested for each material with three water droplets for each sample, and the results are presented as mean ± SD (*n* = 12).

### 2.8 Tensile Strength of Polymers

PTFE, PLA, and PLGA foils were cut into 5 mm × 100 mm rectangle samples. Samples were tested in a universal testing machine using a tensile setup on Instron 3345 with a 50 N load cell. The test was performed according to ASTM standards (D 882-02 and D 638-02a) with a 1.0 mm·min^–1^ crosshead speed until the sample failure. Five independent measurements were carried out for each material, and results of Young’s modulus, tensile strength, and elongation at break are reported as mean ± SD (*n* = 5).

### 2.9 Cytotoxicity Study

L929 cell line was cultured in DMEM medium with 10% (v/v) FBS, 100 U·mL^–1^ penicillin, and 100 μg·mL^–1^ streptomycin in 75 cm^2^ cell culture flasks and maintained at 37 °C in an incubator with 5% CO_2_. The culture was dissociated and divided when the cell reached near full confluency. The cell dissociation protocol was based on a trypsin–EDTA solution procedure. Cell concentrations were counted in the Thoma cell counting chamber (Marienfeld, Germany).

The in vitro cytotoxicity testing methodology was based on the ISO 10993-5 standard protocol. PTFE, PLA, and PLGA discs and discs with PDA patterns and PDA patterns + VAPG were soaked in a 70% solution of EtOH and dried in a laminar flow hood for sterilization. Dry sterile discs were immobilized using a sterile PP insert in a 24-well plate and sunk in 0.5 mL of supplemented DMEM for 24 h. Supplemented DMEM, stored in an incubator for 24 h, was treated as a negative control, and solution DMEM with 1% of Triton X-100 was treated as a positive control. The L929 cell line was maintained in 96-well plates for 24 h in a concentration of 10^5^ cells·mL^–1^ in supplemented DMEM (100 μL of cell suspension per well). After this time, the medium was replaced with disc extracts (n = 5 for each sample) and control media. After 24 h of cultivation with disc extracts and control media, cells were rinsed two times with 100 μL of DPBS and 100 μL of DMEM, without phenol red and supplementation, and 70 μL of XTT solution with electron-coupling reagent solution was added to each culture well and incubated for 4 h. When XTT was reduced to formazan pigment by alive cells, the 100 μL of assay medium from each well was transferred to a new 96-well plate, and the absorbance was measured at 450 nm in a plate spectrophotometer. The cell viability results are reported as mean ± SD (*n* = 5).

### 2.10 Adhesion Study of L929 Murine Cells and Human SMCs

L929 cell line was cultured in DMEM medium with 10% (v/v) FBS, 100 U·mL^–1^ penicillin, and 100 μg·mL^–1^ streptomycin in 75 cm^2^ cell culture flasks and maintained at 37 °C in an incubator with 5% CO_2_. The culture was dissociated and divided when the cell reached near full confluency. The cell dissociation protocol was based on a trypsin–EDTA solution procedure. The cell concentration was counted in the Thoma cell counting chamber. SMCs were cultured in Smooth Muscle Cell Growth Medium 2 with 100 U·mL^–1^ penicillin and 100 μg·mL^–1^ streptomycin in 75 cm^2^ cell culture flasks and maintained at 37 °C in an incubator with 5% CO_2_. The culture was dissociated and divided when the cell reached near full confluency. The cell dissociation protocol was based on a trypsin–EDTA solution procedure. The cell concentration was counted in the Thoma cell counting chamber.

PTFE, PLA, and PLGA discs and discs with PDA patterns and PDA patterns + VAPG (*n* = 2 for each sample) were soaked in a 70% solution of EtOH and dried in a laminar flow hood for sterilization. Sterile PP inserts immobilized dry sterile discs in a 24-well plate. In the case of the L929 cell line, 1 mL of the cell suspension (1 × 10^5^ cells·mL^–1^) in supplemented DMEM was added to each well and kept for 10, 20, and 30 min. In the case of the SMC cell line, 1 mL of the cell suspension (5 × 10^4^ cells·mL^–1^) in supplemented Smooth Muscle Cell Growth Medium 2 was added to each well and kept for 30 min. After each incubation period, the culture medium was removed from the wells, and samples were washed twice with DPBS solution to wash off the culture medium and not adhered cells. Next, samples were soaked in 1 mL of 4% (w/v) DPBS solution of PFA for 15 min, 1 mL of 0.2% (v/v) DPBS solution of Triton X-100 for 5 min, 1 mL of 0.1% DPBS solution of BSA for 1 h, 500 μL of 2.5% DPBS solution of Alexa Fluor 488 Phalloidin for 15 min for cytoskeleton staining, and 500 μL of 300 nM DAPI solution in DPBS for 5 min for nuclei staining; each step was separated by double 5 min washing with 1 mL of DPBS. Prepared samples were transferred on a microscope slide and imaged with a Zeiss LSM 880 confocal laser scanning microscope (CLSM).

### 2.11 Long-Term Culture of SMCs on Substrates

PLA, PLGA, and PTFE discs and discs with PDA patterns and PDA patterns + VAPG (*n* = 4 for each sample) were soaked in a 70% solution of EtOH and dried in a laminar flow hood. Sterile PP inserts immobilized dry sterile discs in a 24-well plate. 1 mL of the cell suspension (1·10^4^ cells·mL^–1^) in supplemented Smooth Muscle Cell Growth Medium 2 was added to each well and kept for 3 and 7 days at 37 °C in an incubator with 5% CO_2_ with culture medium exchanged every 48 h. After each incubation period, the culture medium was removed from the wells, and samples were washed twice with DPBS solution to wash off the culture medium. Next, samples were soaked in 1 mL of 4% (w/v) DPBS solution of PFA for 15 min, 1 mL of 0.2% (v/v) DPBS solution of Triton X-100 for 5 min, 1 mL of 0.1% DPBS solution of BSA for 1 h, 500 μL of 2.5% DPBS solution of Alexa Fluor 488 Phalloidin for 15 min, and 500 μL of 300 nM DAPI solution in DPBS for 5 min; each step was separated by double 5 min washing with 1 mL of DPBS. Prepared samples were transferred on a microscope slide and imaged with a Zeiss LSM 880 CLSM.

### 2.12 Statistical Analysis

The normality of the distribution of PDA patterns size, WCA, and tensile strength properties values was tested using the Kolmogorov–Smirnov test (*p* < 0.05). The difference between the mean values of measured parameters was tested in one-way ANOVA (*p* < 0.05) with post hoc Tukey’s test for multiple comparisons. Statistical analysis was performed using Origin-Pro 8 software (OriginLab Corporation, USA).

## 3 Results and Discussion

**Figure 1:**
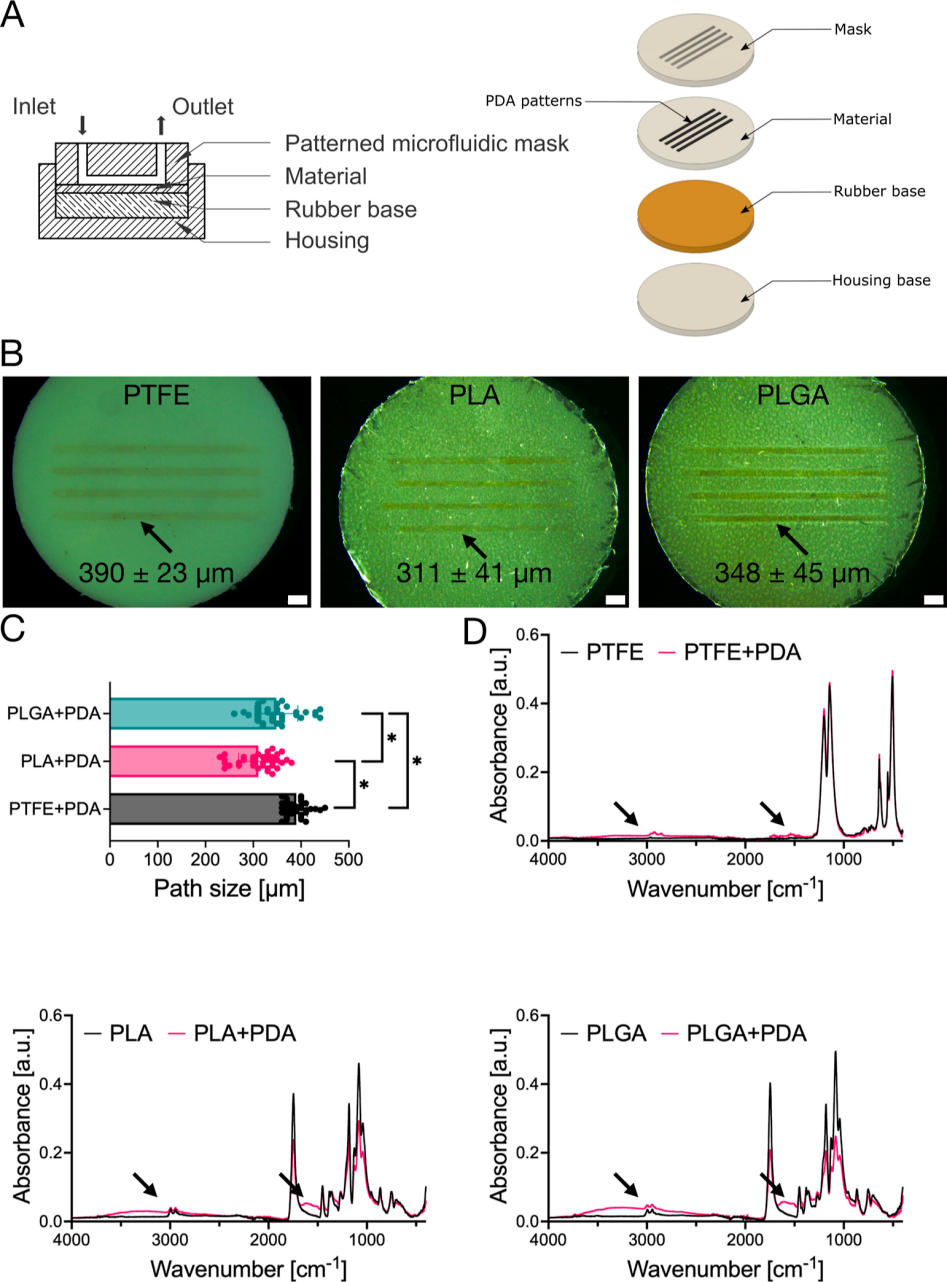
(A) Schematic representation of a 3D-printed device for microfluidic patterning with inlet and outlets for PDA modifying solution and exploded view of the system’s main elements. (B) Stereomicroscopy images of PDA patterns on PTFE, PLA, and PLGA. (C) Comparison of mean PDA path size on PTFE, PLA, and PLGA substrates. (D) FTIR spectra of PTFE, PLA, and PLGA and PDA-modified substrates (PTFE + PDA, PLA + PDA, and PLGA + PDA). Arrows indicate bands characteristic of PDA.

PDA patterns were produced on PTFE, PLA, and PLGA surfaces using the microfluidic device, as shown in Figure 1A. Technical drawings of the device are shown in Figure S1. A 3D-printed microfluidic system allowed us to design patterns of many shapes and mutual arrangements. For demonstration, we also prepared a device capable of producing patterns arranged in a two-letter logo (Figure S2A). Moreover, building a device whose mask is not flat but spatially adapted to a substrate with irregular geometry is possible. In the device prepared for this study, the patterned microfluidic mask contacts the flowing solution, in which dopamine polymerizes, with the surface of the substrate along the designed narrow paths. This contact resulted in brown-black patterns with a size between 300 and 400 μm, depending on the substrate (Figure 1B,C). The FTIR-ATR spectra confirmed that these patterns are composed of PDA. As shown in Figure 1D, the spectra for the patterned regions of substrates contain signals characteristic of PDA: a broad absorption band in the 3700–2800 cm^–1^ region from catechol hydroxyl groups and amino groups and peaks around 1650 and 1550 cm^–1^ from amino groups.^41−43^

Producing PDA patterns using the presented 3D-printed microfluidic system has advantages over previously described methods. Making multiple copies of a microfluidic device in the 3D printing technology for PDA patterning is more straightforward and faster than creating PDMS stamps by the imprinting technology^35^ or fabricating microchannels in PDMS in the molding process.^36^ Using a microfluidic system gives us more control over creating PDA patterns by varying the concentrations of the reagents and the process time. In addition, using periodate as a dopamine oxidizing agent significantly shortens the pattern formation process compared to the classical method of dopamine polymerization by oxidation with atmospheric oxygen in an alkaline environment.^40^

**Figure 2:**
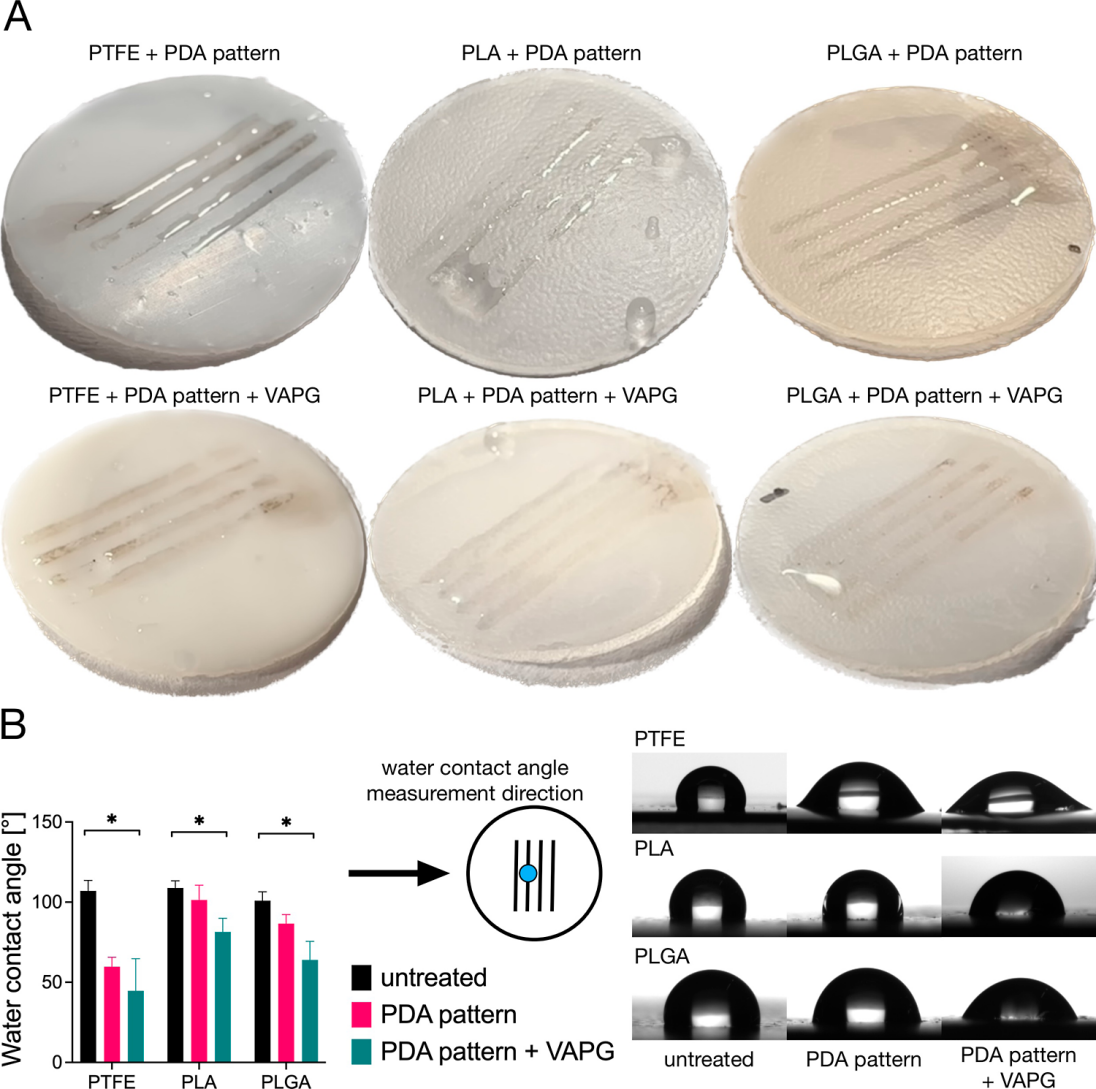
(A) Photographs of wet PTFE, PLA, and PLGA with PDA patterns (first row) and PDA patterns + VAPG (second row). (B) WCA values and photographs of water droplets on the surface of PTFE, PLA, and PLGA untreated materials, PTFE, PLA, and PLGA with PDA patterns, and PTFE, PLA, and PLGA with PDA + VAPG patterns. Asterisk (*) indicates a significant difference (*p* < 0.05) between all three WCA values linked with a bracket.

After a short immersion in water of PDA-patterned substrates, with and without modification with VAPG, we observed the presence of water along the patterns. We did not observe water on the unmodified part of the materials apart from single droplets (Figure 2A). This means that the surface of the substrates was hydrophilized only in places where patterns were produced. As shown in Figure 2B, all tested untreated substrates were hydrophobic with a WCA of 100–110°. The PDA pattern significantly reduced WCA values in patterned regions (p < 0.05) for all substrates. The reduction was the highest for PTFE and PLGA (from 107.0 ± 6.6 to 59.7 ± 5.9° and from 100.9 ± 5.6 to 86.6 ± 5.6°, respectively) and lowest for PLA (from 108.8 ± 4.5 to 101.4 ± 9.2°). The degree of WCA reduction was correlated with the size of the PDA patterns. The reduction was the highest for the broadest patterns formed on PTFE (390 ± 23 μm) and lowest for the narrower patterns on PLGA and PLA (348 ± 45 μm and 311 ± 41 μm, respectively). During the WCA measurement, the water droplets placed on the samples were larger in diameter than the size of the patterns. This means the water droplets were in partial contact with the pattern and the uncoated substrate (Figure 2B). Broader patterns gave a larger contact surface of the droplet with PDA, which led to a higher reduction of the WCA measured. PDA patterns were further functionalized by covalently attaching the VAPG peptide. In the process we used, the VAPG peptide’s terminal primary amino group reacted with the diketone or catechol groups of the PDA via aza-Michael addition and Schiff base reactions.^44^ The attachment of the VAPG peptide to the PDA patterns resulted in a further significant (p < 0.05) reduction of the WCA. The WCA values decreased by about 15 to 23° compared to PDA-patterned substrates before peptide attachment. The results of WCA measurements for PDA-patterned substrates modified with VAPG were characterized by the highest standard deviation values, indicating that the peptide was attached to the PDA layer non-homogeneous.

**Figure 3:**
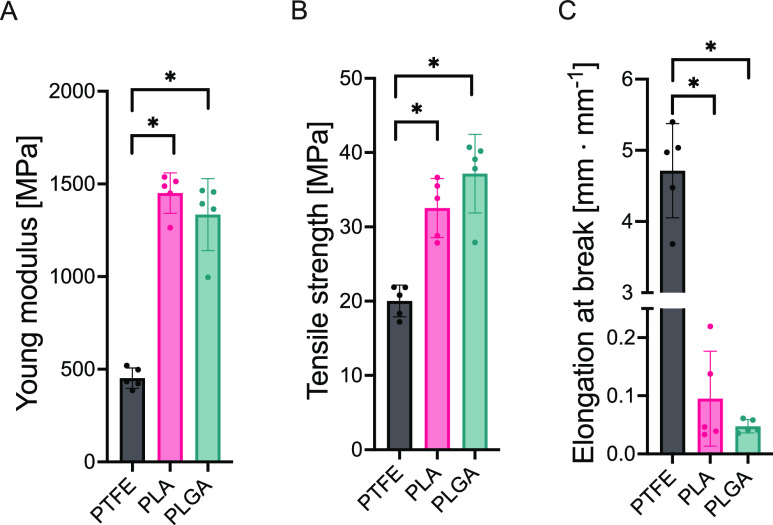
Tensile strength properties of untreated substrates of PTFE, PLA, and PLGA. (A) Young modulus. (B) Tensile strength. (C) Elongation at break. Asterisk (*) indicates a significant difference (*p* < 0.05) between samples linked with a bracket.

As shown in Figure 3, the uniaxial tensile strength measurement showed that the most elastic material tested is PTFE, with a Young modulus of 452.0 MPa. On the other hand, both tested polyesters offered similar rigidity, PLA—1450 MPa, and PLGA—1335 MPa. The Young modulus values corresponded with the elongation at break, where PTFE showed the highest 4.714 mm·mm^–1^ elongation, while PLA and PLGA exhibited low elongation of 0.095 and 0.048 mm·mm^–1^, respectively. The device for PDA pattern fabrication required the polymeric substrate to be pressed into the patterned microfluidic mask. The pressing force was significant enough to deform the substrate. Thus, the most elastic substrate material, PTFE, showed the highest path size (390 ± 23 μm). On the other hand, the stiffest substrate material used in this study, PLA, resulted in the lowest path size (311 ± 23 μm). The presented results indicate that the functionalization of the polymeric materials using the proposed microfluidic device depends not only on the designed size of the pattern on the mask but also on the elastic properties of the substrate.

**Figure 4:**
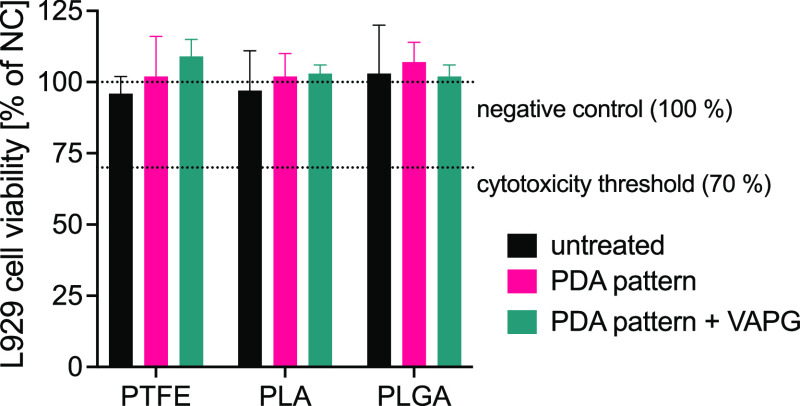
L929 cell viability determined with XTT assay for in vitro cytotoxicity evaluation.

We examined the in vitro cytotoxicity of untreated substrates, PDA-patterned, and PDA-patterned substrates modified with VAPG using the XTT test. Based on the threshold defined by ISO 10993-5 standard, a material is considered cytotoxic when cell viability is less than 70% compared to the negative control. We consider the results of such cytotoxicity measurement as an indicator of the biocompatibility of PDA and PDA + VAPG patterns with cells in vitro. As shown in Figure 4, all tested materials showed cell viability higher than 90%, which means that the substrates of PTFE, PLA, and PLGA are not cytotoxic. Patterns of PDA on the substrate surface neither cause any significant decrease in cell viability nor are PDA-patterned samples modified with VAPG peptide.

**Figure 5:**
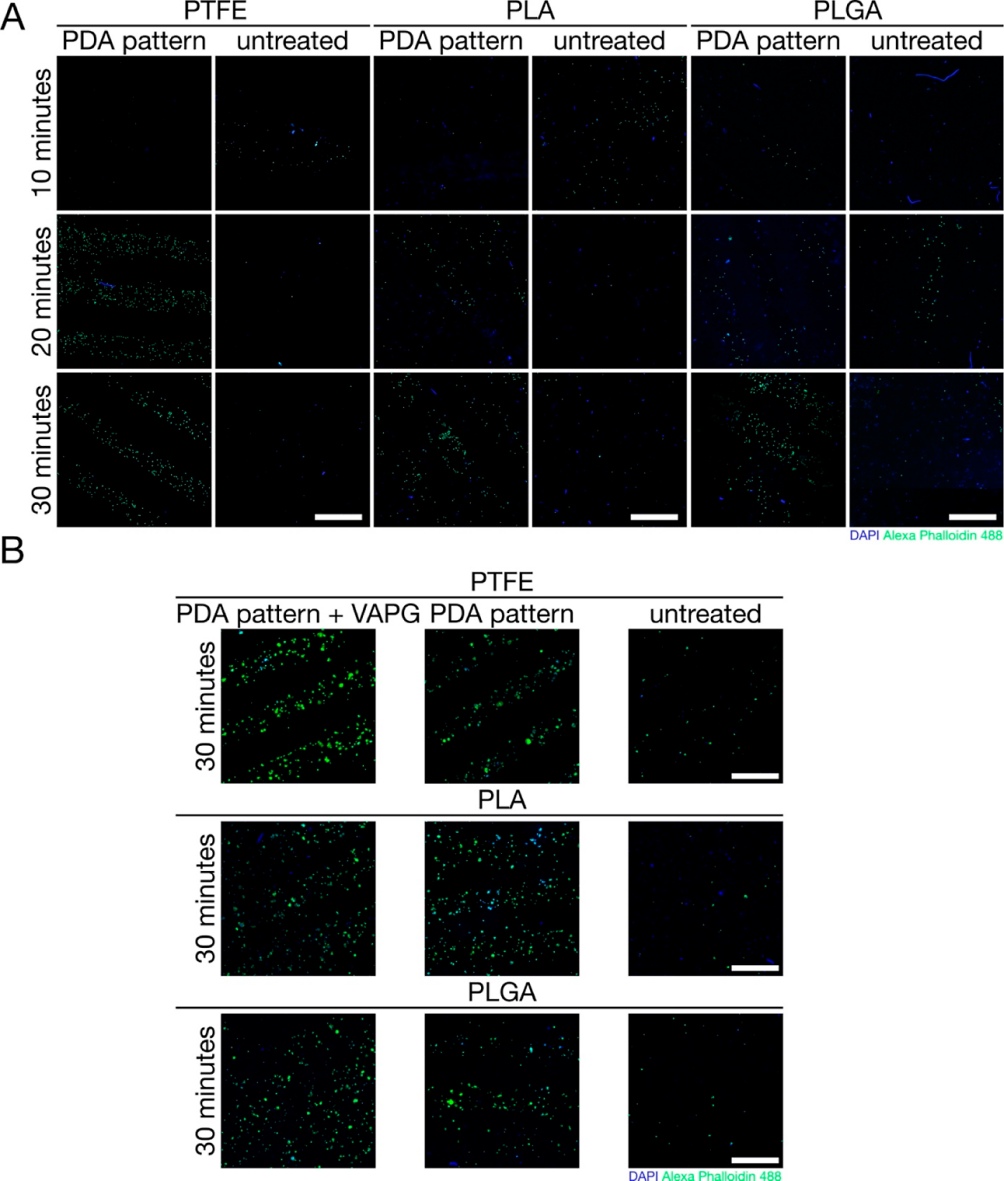
CLSM images showing adhesion study results: (A) L929 cells allowed to adhere over 10, 20, and 30 min to PDA-patterned PTFE, PLA, and PLGA. (B) SMCs allowed to adhere over 30 min to PDA-patterned and PDA-patterned + VAPG PTFE, PLA, and PLGA. Scale bars represent 1 mm.

To further assess the prepared materials, we first examined the adhesion of L929 cells to PDA-patterned materials at the very early stage of culture. As shown in Figure 5A, only single cells with a random distribution were visible on the surface of all tested untreated substrates, even after 30 min of culture. For PDA-patterned PTFE, many cells found only in the PDA patterns were visible after 20 min, and this did not change when the culture lasted for 30 min. In the case of PDA-patterned PLA and PLGA, we observed only single cells after 10 min, but after 20 min, more adherent cells were arranged in striped patterns. After 30 min of cultivation, we observed many cells on PDA patterns and no cells between them for these two substrates. However, the cells were still characterized by a spherical morphology in each tested variant’s test time, suggesting the early stages of the cell attachment process to the substrate. Based on these results, we can conclude that 30 min is a sufficient culture time for the selective adhesion of L929 cells to PDA patterns for all tested substrates.

In the next step, we evaluated whether the designated time is suitable for cultivating SMCs (Figure 5B). This study used PTFE, PLA, and PLGA with PDA patterns and the same group of materials with PDA patterns modified with VAPG peptide. Untreated substrates were used as control samples. After 30 min of this cell line cultivation, we observed randomly distributed single cells on the surface of all tested untreated substrates. Many cells were attached to the surface and arranged along striped patterns for PDA-patterned substrates. For PDA-patterned PLA and PLGA, we observed a small number of cells between the patterns, which did not occur in the case of PDA-patterned PTFE. Similar observations apply to PDA-patterned substrates modified with VAPG, with the number of cells between the stripes for PLA and PLGA patterns being even more remarkable. We can conclude that the covalent attachment of VAPG to PDA patterns did not increase SMC adhesion to the PDA, which was already very good after 30 min of cultivation, even on unmodified PDA patterns. The second conclusion is that VAPG was physically attached to the surface of PLA and PLGA and was not completely washed off by rinsing with water. VAPG physically adhered to the polyesters probably causes an increase in the number of cells adhered between the pattern stripes for these substrates. This phenomenon does not occur for PTFE, probably due to its low surface energy causing its resistance to protein adsorption and cell adhesion.^38^

**Figure 6:**
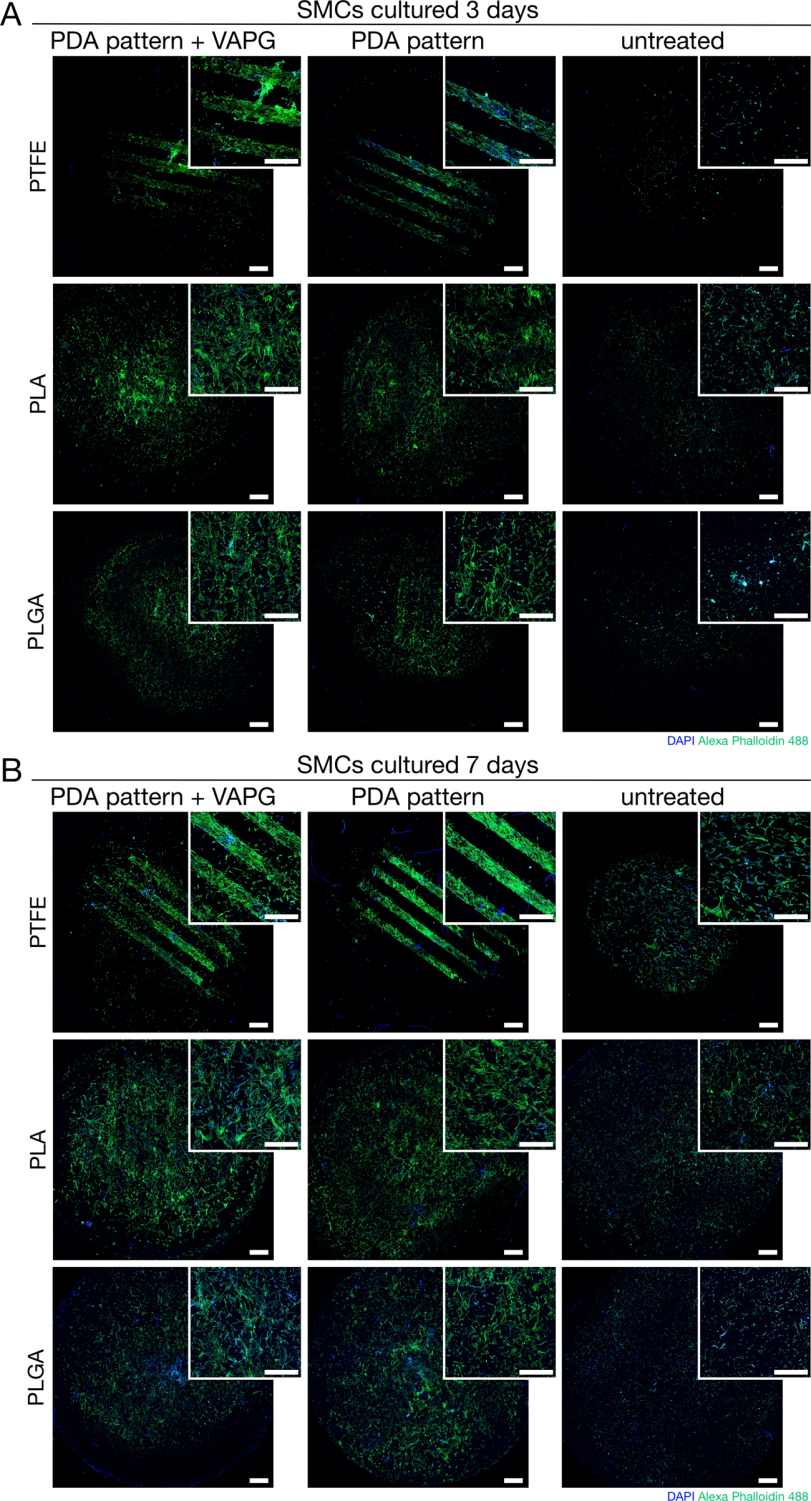
CLSM images showing results of (A) 3 days-long culture of SMCs on the surface of PDA- and PDA + VAPG-patterned PTFE, PLA, and PLGA; (B) 7 days-long culture of SMCs on the surface of PDA- and PDA + VAPG-patterned PTFE, PLA, and PLGA. Scale bars represent 1 mm.

The long-term culture of SMCs was started with cell adhesion for 30 min, as determined in the previous experiment. After 30 min, the medium with unadhered cells was replaced with a fresh one. After 3 and 7 days of cell culture, cell growth on the surface of the tested materials was imaged by CLSM, and the results are presented in Figure 6. The SMCs showed poor growth on the surface of the untreated materials after 3 days of cultivation (Figure 6A). We found single non-flattened cells on the surface of PTFE and PLGA. Only PLA showed a single place of accumulation of cells with a tendency to flatten. On day 7 (Figure 6B), we observed more cells on the surface of untreated substrates, but they still did not form a homogeneous layer, and only some cells were flattened. The most significant gaps between the cells were visible for PTFE and the smallest for PLA. We observed that SMCs only proliferated along the stripe patterns for PDA-patterned PTFE. After 3 days, the patterns were mostly overgrown with flattened cells, and after 7 days, the cells formed a uniform layer along the boundaries defined by the PDA patterns. This was similar for PDA-patterned PTFE modified with VAPG. Moreover, cell growth outside the stripe patterns was observed in this case, especially after 7 days of culture. We had similar observations for the long-term cultivation of SMCs on PTFE with PDA patterns formed in the PW logo (Figure S2B).

We saw a different situation for PDA-patterned PLA and PLGA and the same substrates with PDA patterns modified with VAPG. After 3 days of cultivation on these materials, we observed that the SMCs had proliferated on the surface of the entire samples. Cells growing along the stripe patterns were noticeable, but growth was not stopped within the limits of the patterns. However, unlike untreated materials, we saw many flattened cells on the surface. This means that cells found a favorable environment to adhere to and proliferate on the surface of the patterns. Still, then they grew freely in all possible directions throughout the material. After 7 days, the situation was similar, with much more cells over the entire surface of the materials. We did not observe a significant difference between substrates with PDA patterns only and those additionally modified with VAPG.

Results from the long-term culture of SMCs indicated that PDA patterns allowed for selective cell adhesion and patterned cell growth on materials resistant to cell adhesion and proliferation, like PTFE, but were ineffective on materials susceptible to cell adhesion, like PLA and PLGA. PDA itself promoted cell adhesion and proliferation so effectively that additional attachment of the adhesion peptide was unnecessary. As PDA is not a selective promoter of adhesion and proliferation for one cell type, we speculate that our system could also be applied to the patterned growth of non-muscle cells. Other authors have successfully applied topographic and chemical micropatterning to patterned growth of nerve cells.^39,45−50^ The effectiveness of our 3D-printed microfluidic system in culturing cells of this type should be the subject of future research.

## 4 Conclusions

The present study describes the application of a 3D-printed microfluidic device for producing PDA patterns on the surface of materials for tissue engineering and regenerative medicine: PTFE, PLA, and PLGA. This type of device can be easily customized to fabricate patterns of various shapes and sizes, and the geometry of the interface mask can be adapted to the profile of the material to be patterned, which may not be flat. Because coated polymers vary in mechanical properties, we determined that the broadest paths in the pattern are produced on the softest material.

We covalently bonded the SMC adhesion-promoting VAPG peptide to the created PDA patterns. The surface of the substrates was hydrophilized in the places where PDA patterns were formed, and the attachment of VAPG enhanced this effect. The WCA value reduction was more significant for broader PDA. All PDA-patterned substrates and substrates with PDA patterns modified with VAPG showed no cytotoxicity in the XTT assay, where cell viability was greater than 90% for all variants tested.

PDA patterns promoted the selective adhesion of L929 cells on the surface of PTFE, PLA, and PLGA. The adhered cells were arranged in striped patterns. Many cells were found almost only in the PDA patterns and were visible after 20 min of adhesion time for PDA-patterned PTFE and after 30 min for PDA-patterned PLA and PLGA. SMCs also adhered to the materials over 30 min; however, in this case, we used PDA-patterned substrates with and without a covalently attached VAPG peptide. When the substrate was PTFE, adhesion was selective, and SMCs were present only on the surface of the PDA patterns. In the case of PLA and PLGA, some cells were also present between the pattern stripes. In general, covalent attachment of VAPG to PDA patterns did not increase SMC adhesion when the cultivation time was 30 min.

PDA patterns also promote SMC proliferation in long-term culture. After 7 days of cultivation, we observed the proliferation of cells only along the stripe patterns on PTFE. With and without VAPG attached, the PDA patterns on PTFE were entirely overgrown with flattened cells. In the case of PLA and PLGA, with and without VAPG attached, the SMCs adhered and proliferated on the pattern’s surface but, over a longer time, grew in all possible directions throughout the material. These results show that PDA patterns allow for selective cell adhesion and patterned cell growth. Still, these advantages benefit application to materials resistant to cell adhesion and proliferation, like PTFE. The additional attachment of the VAPG peptide to the PDA patterns did not bring measurable benefits due to the high increase in adhesion and patterned cell proliferation by PDA itself. This observation is significant because it may reduce cost (the high cost of cell adhesion-promoting peptides) and shorten the surface modification process (by cutting the peptide modification step).

We confirmed the thesis that PDA can be used in systems that aim to create patterns on polymers that will support SMC adhesion and growth. The advantages of a 3D-printed microfluidic micropattern device and the properties of PDA to promote cell adhesion in our system can be used for patterned SMC growth on certain materials used in tissue engineering and regenerative medicine. The suitability of our system for patterned growth of other cells naturally forming aligned tissues, e.g., nerve cells, requires further investigation.

## Supporting Information Available

Technical drawing of the presented microfluidic device; photographs of the PDA patterned PW (Politechnika Warszawska in Polish) Warsaw University of Technology logo on the PTFE substrate, and CLSM images of SMCs cultured for 7 days on such substrates (PDF)

## Abbreviations

ATR: attenuated total reflection
ATR-FTIR: attenuated total reflectance-Fourier transform infrared
BSA: bovine serum albumin
CLSM: confocal laser scanning microscope
DAPI: 4′,6-diamidino-2-phenylindole dihydrochloride
DMEM: Dulbecco’s modified Eagle’s medium
DPBS: Dulbecco’s phosphate-buffered saline
DRG: dorsal root ganglion
FBS: fetal bovine serum
GelMA: gelatin methacryloyl
hNSCs: human primary adult neural stem cells
MTG: microbial transglutaminase
NGF: nerve growth factor
PDA: polydopamine
PDMS: polydimethylsiloxane
PEG: poly(ethylene glycol)
PFA: paraformaldehyde
PKSPMA: poly(potassium 3-sulfopropyl methacrylate)
PLA: poly(l-lactic acid-*co*-D,l-lactic acid)
PLGA: poly(lactic acid-*co*-glycolic acid)
PP: polypropylene
PTFE: polytetrafluoroethylene
PW: Warsaw University of Technology (Politechnika Warszawska in Polish) logo
SCs: Schwann cells
SMCs: smooth muscle cells
VAPG: Val-Ala-Pro-Gly peptide
WCA: water contact angle
XTT: CyQUANT XTT Cell Viability Assay.
