# Supplementary material for: System for Patterning Polydopamine and VAPG Peptide on Polytetrafluoroethylene and Biodegradable Polyesters for Patterned Growth of Smooth Muscle Cells In Vitro
Source: ACS Omega. 2023 Jun 5;8(24):22055–66. doi: 10.1021/acsomega.3c02114 (PMC10285958; doi:10.1021/acsomega.3c02114)
Supplement: Supplementary file 1 — ao3c02114_si_001.pdf [file ao3c02114_si_001.pdf]

Supplementary materials for:

System for Patterning Polydopamine and VAPG Peptide on Polytetrafluoroethylene and Biodegradable Polyesters for Patterned Growth of Smooth Muscle Cells In Vitro

Kamil Kopeć[1], Rafał Podgórski[1], Tomasz Ciach[1,2], and Michał Wojasiński[1,*]

[1]Warsaw University of Technology, Faculty of Chemical and Process Engineering, Department of Biotechnology and Bioprocess Engineering, Waryńskiego 1, 00-645 Warsaw, Poland

[2]Warsaw University of Technology, CEZAMAT, Poleczki 19, 02-822 Warsaw, Poland

*Corresponding author: michal.wojasinski@pw.edu.pl

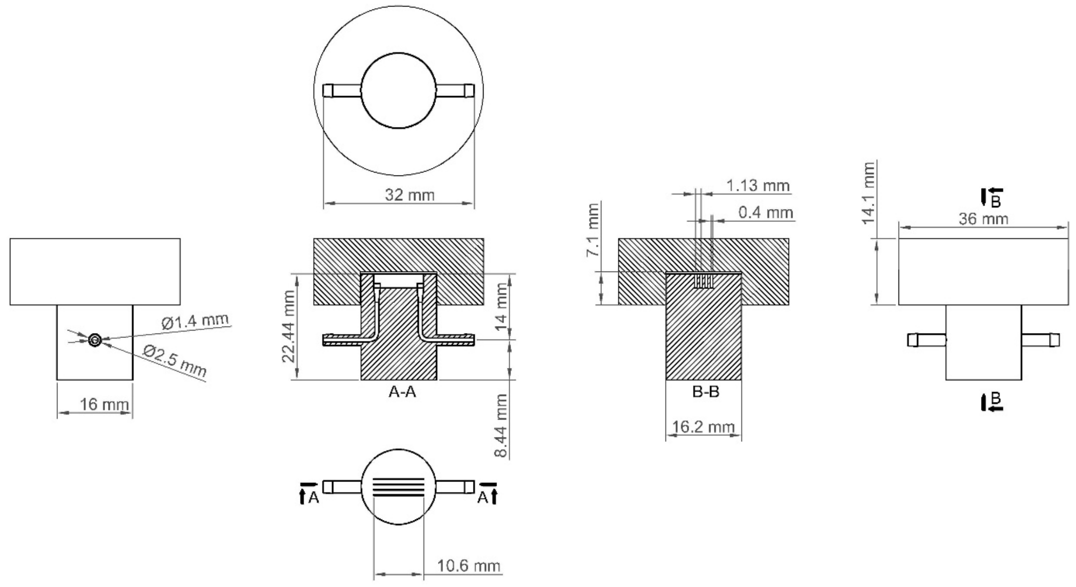

Figure S1. Detailed design of the device used for patterning PDA on PTFE, PLA, and PLGA for further VAPG functionalization.

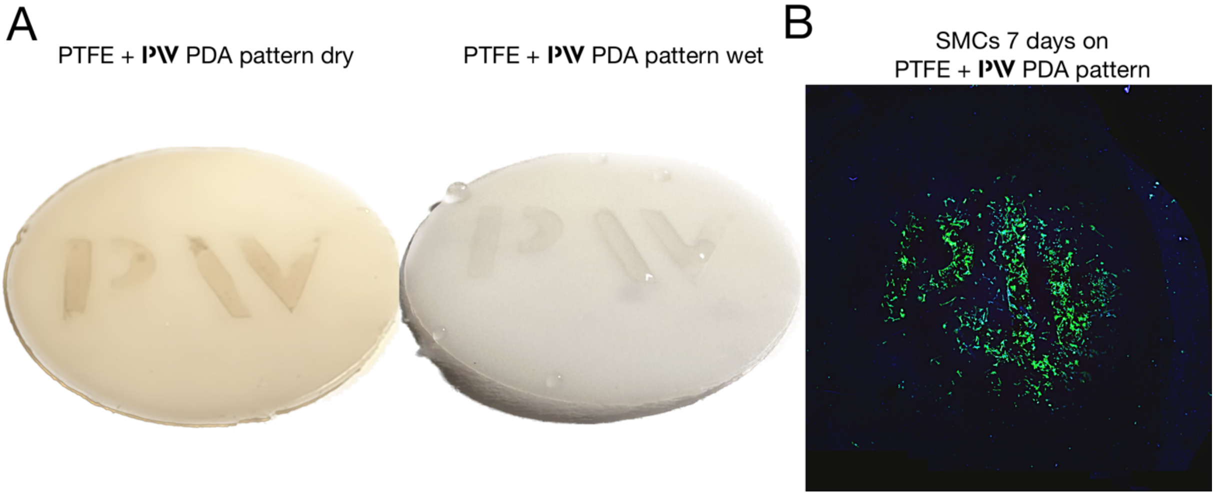

Figure S2. A – photographs of the PTFE surface with the PW logo: dry and wetted. B – CLSM image of SMCs cultured over 7 days on the surface of PTFE with a PDA pattern in the shape of the PW logo.
